# Editorial: Origin and evolution of hepatitis viruses, volume II

**DOI:** 10.3389/fmicb.2023.1241705

**Published:** 2023-07-11

**Authors:** Avik Biswas, Sibnarayan Datta

**Affiliations:** ^1^Department of Signal Transduction and Biogenic Amines, Chittaranjan National Cancer Institute (CNCI), Kolkata, India; ^2^Molecular Virology Laboratory, Entomology & Biothreat Management Division, Defence Research Laboratory, Defence R&D Organization (DRDO), Tezpur, Assam, India

**Keywords:** HBV, evolution, genotypes, subgenotypes, molecular clock

Hepatitis B virus (HBV) is one of the prototype members of the *Hepadnaviridae* family, which is the causative agent of the most prevalent infection worldwide. Globally, around 300 million people are chronically infected with HBV, with substantial mortality from cirrhosis and Hepatocellular carcinoma (HCC) through multimodal interaction with the host (Li et al., 2019; Kar et al., 2023). Despite the availability of safe and effective vaccine and direct acting antiviral therapies for HBV treatment, new infections and complete cure is yet to be achieved. One of the key reasons behind this might be the high mutation rate in the HBV genome, which confers it with a considerable plasticity in terms of its propagation and pathogenicity. Based on analyses of complete genome sequences, HBV has been phylogenetically classified into at least ten genotypes (A–J). Based on the intra-genotype diversity of HBV, multiple subgenotypes with moderate differences in genome sequence have been denoted. The spread of HBV is believed to occur through human-to-human transmission, and involvement of any non-human or inanimate reservoirs remains undiscovered. The geographical correlation of HBV genotypes with the highly acclaimed patterns of historic events of human migrations further establishes the co-evolution of the HBV genome with its human hosts (Datta, 2020). Incidentally, the HBV genomic mutations which are associated with clinical outcomes also vary among different genotypes of HBV as well as differences in environmental factors associated with viral persistence and pathogenesis. The current topic highlights the evolutionary aspects of specific HBV genotypes/subgenotypes, their distribution patterns in distinct geographical locations as well as serological prevalence in relation to socioeconomic strata.

Since its discovery, a huge volume of scientific literature has been generated, which primarily focus on the biology, epidemiology, and pathology of HBV. In sharp comparison, research on origin and evolution of Hepatitis viruses, including HBV is very limited. Especially, for HBV, different researchers have attempted to estimate its origin and distribution of different genotypes/subgenotypes across the globe, using different mathematical/statistical models. However, these studies have resulted in extremely wide variable time-scales, ranging from few 100 years to some 1,000 years. With the unprecedented advancements in the fields of genome sequencing and analyses methodologies, ancient HBV (aHBV) genomes have recently been characterized from prehistoric archeological remains, which has established the long association of HBV with humans (Krause-Kyora et al., 2018; Mühlemann et al., 2018; Spyrou et al., 2019; Kocher et al., 2021). Considering the well-established fact of clinical difference between different HBV genotypes/subgenotypes, studies on the origin and evolution has become more relevant from the perspective of global control of the virus and the disease burden caused by it.

In this issue, using molecular clock analyses, Jose-Abrego et al. estimated the ancestral divergence times/events of the new- world HBV genotypes F and H. The study further highlighted the diversification time points of subgenotypes of HBV H (H1 to H4). Such clock analyses highlight the selective spread of one genotype over the other and reconfigures the viral phylogeny. In another study, Araujo and Osiowy envisioned HBV G from its origin to current day evolutionary status. The comprehensive review describes the unique features of HBV G which makes it different from its neighboring members. Unlike other genotypes, this has some peculiar alterations in its genome and hence the encoded proteins. The genome exhibits 36-nt insertion after the fifth nucleotide following the core translation initiation point along with 2 translational stop codons that prevents the expression of hepatitis B e antigen (HBeAg), which is essential for the establishment of infection and immune evasion. Due to the lack of this protein, the virus seems to establish co-infection with the other HBV genotypes. Interestingly among the human HBV genotypes of clinical significance at present HBV G seems to occupy a phylogenetically distinct cluster.

Study by Kim et al. reported the prevalence of C2(3) clade among C2 subgenotypes in South Korea. HBV endemic East-Asian countries like China, Japan and South Korea persists infection from HBV subgenotype C2. Of these, genotype C is mainly distributed in Asia, is the largest group and comprises more than seven subgenotypes (C1–C7). Subgenotype C2 forms three different phylogenetic clades, C2(1), C2(2), and C2(3), and causes the highest number of genotype C infections in the major HBV endemic countries, namely China, Japan, and South Korea. Epidemiological studies have demonstrated that in far eastern regions, the high incidences of HBV related HCC are more often associated with HBV genotype C. Nevertheless, the distribution patterns and unique mutations and polymorphisms associated with this subgenotype reported in the study bears substantial clinical relevance and will add useful information to the existing literature.

India records among the highest-ranking countries in terms of tribal population and Odisha which lies in the South-eastern part of India comprises the maximum number of scheduled tribes. Bhattacharya et al. did a descriptive study on the HBsAg prevalence among the tribal and particularly vulnerable tribal populations of Odisha. Their study revealed that few of them showed a higher prevalence to HBV infection despite sharing similar geographical location, attributes and other socio-cultural aspects. Although various vaccination programs have been conducted in these remote areas to prevent the spread of the infection, the maximum population still behaves non-immunized due to the unresponsiveness of the vaccines, which might be a cumulative effect of the high rate of mutation in the HBV genome resulting in vaccine escape like phenomena.

## Author contributions

Editorial draft prepared by AB and SD. All authors contributed to the article and approved the submitted version.
